# Predictive value of bowel dose-volume for severe radiation-induced lymphopenia and survival in cervical cancer

**DOI:** 10.3389/fimmu.2024.1459206

**Published:** 2024-11-01

**Authors:** Jingjing Li, Qingqing Chen, Zhengcao Liu, Yingying Xu, Shengjun Ji

**Affiliations:** ^1^ Department of Radiotherapy, the Affiliated Suzhou Hospital of Nanjing Medical University, Suzhou, China; ^2^ Department of Radiotherapy, the Second Affiliated Hospital of Soochow University, Suzhou, China

**Keywords:** cervical cancer, radiation-induced lymphopenia, bowel, dose-volume, prognosis

## Abstract

**Background:**

Radiation-induced lymphopenia (RIL) is closely related to the prognosis of cervical cancer patients and may affect the efficacy of immune checkpoint inhibitors (ICIs). However, the factors influencing RIL are not very clear. In addition to bone marrow (BM) dose-volume, animal studies indicate radiation-induced bowel injury may be a more crucial factor. Further clarification of the correlation between RIL and bowel dose-volume is important for cervical cancer treatment.

**Methods:**

Cervical cancer patients treated with postoperative radiotherapy or radical radiotherapy were eligible for this retrospective study. Clinical characteristics, dose parameters of bowel and BM, planning target volume (PTV) size, overall survival (OS) and progression-free survival (PFS) were recorded. The absolute lymphocyte count<0.5×10^9^/L at radiotherapy end was defined as severe RIL (sRIL). Hazard ratio (HR) and 95% confidence interval (Cl)were estimated using Cox regression models. Survival curve was plotted using the Kaplan-Meier method. On this basis, the receiver operating characteristics (ROC) curve was used to calculate the area under the curve (AUC) for radiation parameters with sRIL as the state variable.

**Result:**

A total of 118 cervical cancer patients were included in this study, with a median follow-up time of 57.6 months. In multivariable Cox regression analysis, international Federation of Gynecology and obstetrics (FIGO) stage (HR, 11.806; 95% CI, 3.256-42.809; p<0.001), concurrent chemotherapy (HR, 0.200; 95% CI, 0.054-0.748; p=0.017), sRIL after radiotherapy (HR, 6.009; 95% CI, 1.361-26.539; p=0.018), and pathological type (HR, 2.261; 95% CI, 1.043-4.901; p=0.039) were significantly correlated with OS. Patients with sRIL had significantly decreased OS (79.1% vs 94.1%; HR, 3.81; 95%CI, 1.46-9.92; p=0.023). In binary logistic regression analysis, sRIL was significantly correlated with bowel V45 (Odds radio (OR), 1.025; 95%CI, 1.007-1.044; p=0.007), BM V10 (OR, 0.987; 95%CI, 0.978-0.997; p=0.011), BM V20 (OR, 1.017; 95%CI, 1.002-1.031, p=0.027), and PTV size (OR, 0.998; 95%CI, 0.996-1.000; p=0.026). The ROC curve showed, bowel V45 (AUC=0.787, p<0.001) was the best indicator for predicting sRIL.

**Conclusion:**

SRIL after radiotherapy could significantly predict decreased OS. In addition, sRIL is associated with higher bowel, BM dose-volume, PTV size, indicating that the bowel may be an important organ leading to an increased risk of sRIL.

## 1 Introduction

Lymphocyte-led immunity plays a pivotal role in the occurrence and progression of tumors. To date, radiation-induced lymphopenia (RIL) is identified in numerous solid tumors, and has been confirmed to correlate with poor prognoses of esophageal cancer, lung cancer, colorectal cancer, and so on (1–4). In addition, RIL may negatively impact the effectiveness of immunotherapy (2, 5). A study has found that the number of CD4+ and PD-1+T cells in peripheral blood decreased and inhibitory regulatory T cells increased after concurrent chemoradiotherapy (CCRT) in cervical cancer (CC), indicating that CCRT may have a negative impact on peripheral blood T cell immunity (6). The precise mechanisms driving RIL remain elusive, though it is postulated that irradiation of circulating blood and bone marrow (BM) may contribute to its development (2).

Immunotherapy has become an important treatment strategy in locally advanced, recurrent and metastatic cervical cancer (7). The KEYNOTE-A18 showed that compared to the control group, pembrolizumab combined with CCRT significantly improved progression-free survival (PFS) in locally advanced CC patients (8). Notably, 89% of CC patients experienced absolute lymphocyte count (ALC) ≤ 500 cells/mm^3^ during CCRT, a rate significantly higher than that observed in other solid tumors (9). Consequently, comprehensive research into the correlation between RIL, prognosis, and associated factors may offer valuable insights for optimizing CC treatment strategies.

In recent years, there have been many studies on the factors that affect sRIL in CC radiotherapy, including BM dose-volume constraints, chemotherapy, bowel factors, and more advanced radiotherapy technique. The BM dose-volume constraints have been widely recognized, but related studies still have contradictions. While numerous studies have investigated the correlation between hematologic toxicity and bone marrow sparing (BMS), only six articles have specifically focused on lymphocytes (10–15). Although some studies indicate that an increase in BM dose-volume is significantly correlated with a decrease in lymphocytes (15, 16), this correlation has been contradicted by two prospective controlled studies (10, 14). Yang L et al. found that early onset of severe lymphopenia during definitive RT was associated with mean body dose and predicted poor survival in CC patients (17). Animal studies have shown that radiation-induced bowel injury can cause significant apoptosis of peripheral blood lymphocytes. Specifically, irradiating the abdomen of mice with ultra-high dose rate or conventional dose rate protons significantly decreased the total lymphocyte count in the peripheral blood within 24 hours (18). Additionally, research has demonstrated that if BMS is overly emphasized in CC radiotherapy, it can significantly increase the dose to other organs at risk (OAR), such as bowel (19, 20). Consequently, we propose that an increase in bowel dose-volume during radiotherapy may reduce ALC, thereby affecting the prognosis of CC patients. Therefore, this study aims to explore the prognostic significance of sRIL and the potential impact of increased bowel dose-volume. The research results may provide references for clinicians in treatment decision-making.

## 2 Materials and methods

### 2.1 Patient selection

We conducted a retrospective cohort study of patients who 1) received external beam radiation therapy (EBRT) from 2015 to 2020 at Affiliated Suzhou Hospital with Nanjing Medical University; 2) had histologically confirmed cervical cancer; 3) received radical radiotherapy or postoperative EBRT, combined with or without platinum based concurrent chemotherapy; 4) had ALC before and after RT within one week. We excluded patients with a second primary tumor or incomplete follow-up information. This research was approved by the Ethics Committee of the Affiliated Suzhou Hospital with Nanjing Medical University.

### 2.2 Data collection

The following clinical characteristics were obtained: age, pathological type, FIGO stage, radiotherapy technique, concurrent chemotherapy. ALC were recorded within one week before and after radiotherapy. According to the dose volume histogram, we calculated planning target volume (PTV) size, the relative volumes of bowel receiving 5, 10, 15, 30, 35, 40 and 45 Gy (bowel V5, V10, V15, V30, V35, V40, and V45) and collected BM V10, V20, V30, V40 using the same method. According to the Common Terminology Criteria for Adverse Events (version 5.0), lymphocyte count after radiotherapy were classified, and grade 3 and 4 lymphocyte reduction were defined as ALC < 0.5-0.2×10^9^/L and < 0.2×10^9^/L, respectively. Those who experienced grade 3 and 4 lymphocyte reduction after radiotherapy were defined as sRIL. PFS was calculated from the start date of radiotherapy to the first occurrence of disease progression or death due to any reason. Overall survival (OS) referred to the survival date from the start of radiotherapy to death due to any cause or final follow-up confirmation.

### 2.3 Treatment and follow-up

Radiotherapy was conducted using a Varian linear accelerator (TrueBeamSN 1587, USA). All patients used two types of EBRT techniques, volumetric modulated arc therapy (VMAT) or intensity modulated radiation therapy (IMRT). Delineation of the target area for postoperative radiotherapy of CC: clinical target volume (CTV): pelvic lymph node drainage area (common iliac, external iliac, internal iliac, presacral, obturator region) and parametrial/vaginal tissue, partial vagina; PTV: CTV expanded 0.7 cm outward in all directions. Radical radiotherapy: gross tumor volume (GTV)-T: primary tumor; CTV included GTV-T, the cervix and uterine body, and parametrial/vaginal tissue, parametrial fat, ovaries, and partial vagina, and pelvic lymph node drainage area (common iliac, external iliac, internal iliac, presacral, obturator region); PTV were defined as CTV with 7mm margins; OAR mainly delineated the bowel, bladder, femoral head, and rectum. The bowel target area was defined as the line connecting the outermost edges of small intestine and colon within PTV and 2 cm above PTV, including the lumen, intestinal tissue, and most of mesentery (21). OAR limits included bladder D50 ≤ 50Gy, bowel D10 ≤ 50Gy, femoral head D5 ≤ 50Gy, and rectum D50 ≤ 50Gy. EBRT: 45-50Gy/25F, 5 times a week. Positive lymph node received concurrent radiotherapy with an additional dose of 10-15Gy. Brachytherapy was performed under CT guidance and started 20F after EBRT, using high-dose iridium. The treatment frequency was once a day, alternating with EBRT, and 6-7 Gy each time for a total of 4-5 treatments.

Concurrent chemotherapy, based on platinum, was considered by the attending physician according to the patients’ age and the presence of high-risk factors. If the patient experienced serious complications from radiotherapy or chemotherapy, the attending physician decided to interrupt the chemotherapy once at their discretion. After radiotherapy, patients were followed up every 3 months for the first 2 years, every 6 months for 3 to 5 years, and annually thereafter. Surveillance evaluations include tumor markers, physical and imaging examinations, such as chest and abdominal CT or PET-CT, pelvic MRI, and biopsy if necessary.

### 2.4 Statistical analysis

The median and inter-quartile range (IQR) were used to sum up continuous variables. Categorical variables were descriptively analyzed using proportion and frequency. Multivariable analysis used the Cox regression models to determine the hazard ratio (HR) and 95% confidence interval (Cl) in order to evaluate the relevance between multiple potential factors and PFS, OS. We used binary logistic regression to analyze the correlation between sRIL and clinical features, dose-volume parameters, and to identify the risk factors affecting sRIL. On this basis, the receiver operating characteristics (ROC) curve was used to, with sRIL as the state variable, determine the area under the curve (AUC) to evaluate predictive effectiveness. SPSS 26.0 was used for data analysis. A p-value less than 0.05 was considered statistically significant.

## 3 Results

### 3.1 Patient characteristics

This study included 118 patients with CC. The clinical characteristics and dose-volume parameters are detailed in **Table 1**. The median follow-up time was 57.6 months, and median age was 52 years. A total of 105 (89.0%) patients had squamous cell carcinoma, and FIGO stage (2018) distribution of locally advanced stage (IIB-IVA) was 38 (32.2%). The main radiotherapy technique was IMRT, and 78 (66.1%) patients received IMRT while 40 (33.9%) received VMAT. The median values of ALC before and after radiotherapy were 1.4 × 10^9^/L and 0.4 × 10^9^/L, respectively. Among them, 100 (84.7%) patients received CCRT and 67 (56.8%) patients experienced severe lymphocyte reduction after radiotherapy.

**Table 1:** Patient characteristics and clinical outcomes.

Characteristics	N (%) or Median (IQR)
Age (years)	52 (44-60)
Pathology	
Squamous cell carcinoma	105 (89.0%)
Adenocarcinoma	8 (6.8%)
other	5 (4.2%)
FIGO stage (2018)	
I-IIA	80 (67.8%)
IIB-IVA	38 (32.2%)
EBRT technique	
IMRT	78 (66.1%)
VMAT	40 (33.9%)
Chemoradiotherapy	
Yes	100 (84.7%)
No	18 (15.3%)
Bowel V5	1104.6 (843.0-1305.3)
Bowel V10	1016.5 (807.3-1239.2)
Bowel V15	940.1 (720.7-1179.4)
Bowel V30	508.2 (326.2-666.6)
Bowel V35	337.0 (227.5-517.2)
Bowel V40	248.8 (150.3-385.6)
Bowel V45	153.0 (90.9-272.1)
BM V10	968.2 (884.1-1113.1)
BM V20	861.9 (770.2-964.3)
BM V30	570.8 (483.9-662.5)
BM V40	321.8 (256.1-393.7)
PTV size	1237.8 (955.5-1542.9)
Pre-RT ALC (cells ×109/L)	1.40 (1.10-1.70)
Post-RT ALC (cells ×109/L)	0.40 (0.30-0.50)
Grade of post-RT lymphopenia	
0	4 (3.3%)
1	2 (1.7%)
2	45 (38.2%)
3	66 (56.0%)
4	1 (0.8%)
mPFS (months)	54.3 (46.6-79.3)
mOS (months)	57.6 (47.5-92.8)
Disease progression	
Yes	22 (18.6%)
No	96 (81.4%)
Death	
Yes	17 (14.4%)
No	101 (85.6%)

### 3.2 sRIL and survival

Under univariable analysis, the following factors were associated with OS: FIGO stage (HR, 12.339; 95%CI, 3.320-45.859; p<0.001), concurrent chemotherapy (HR, 0.202; 95%CI, 0.046-0.886; p=0.034), sRIL (HR, 6.846; 95%CI, 1.427-32.854; p=0.016).

Multivariable Cox regression analysis indicated that sRIL was independently significant for decreased OS (HR,6.009; 95%CI, 1.361-26.539; p=0.018). Other adjusting factors including pathological type (HR, 2.261; 95% CI, 1.043-4.901; p=0.039), concurrent chemotherapy (HR, 0.200; 95% CI, 0.054-0.748; p=0.017), and FIGO stage (HR, 11.806; 95% CI, 3.256-42.809; p<0.001) were also predictive of OS (**Table 2**).

**Table 2:** Univariable and multivariable Cox regression analysis for overall survival.

Variable	Univariable analysis		Multivariable analysis	
	HR (95%CI)	p value	HR (95%CI)	p value
Age	0.996 (0.942,1.053)	0.885		
Pathological type	2.460 (0.995,6.083)	0.051	2.261 (1.043,4.901)	0.039
FIGO stage (I-II/III-IV)	12.339 (3.320,45.859)	<0.001	11.806 (3.256,42.809)	<0.001
EBRT technique (IMRT/VMAT)	0.781 (0.236,2.583)	0.685		
Chemoradiotherapy (Yes or not)	0.202 (0.046,0.886)	0.034	0.200 (0.054,0.748)	0.017
Post-RT sRIL	6.846 (1.427,32.854)	0.016	6.009 (1.361,26.539)	0.018
PTV size	1.000 (0.999,1.001)	0.483		

**Figure 1:**
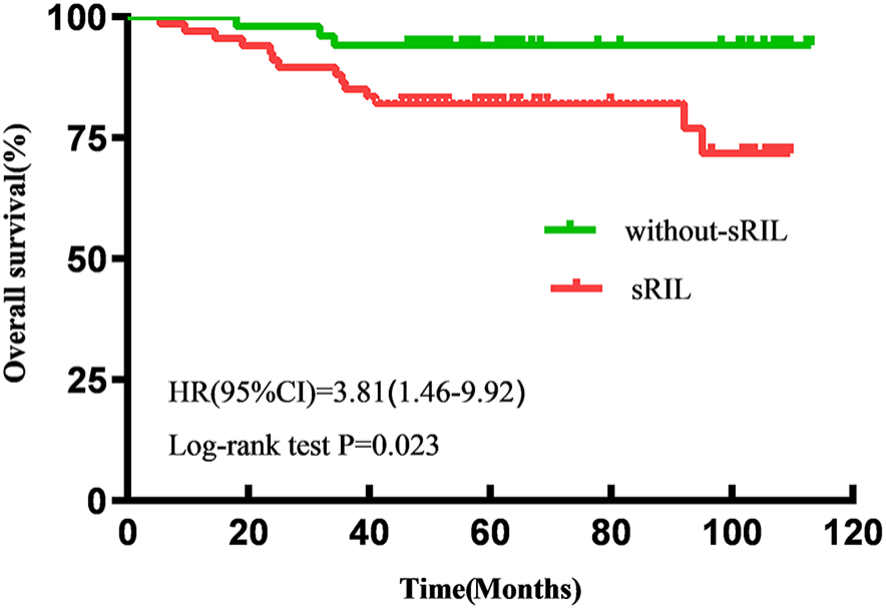
Kaplan-Meier curves of overall survival.

**Figure 2:**
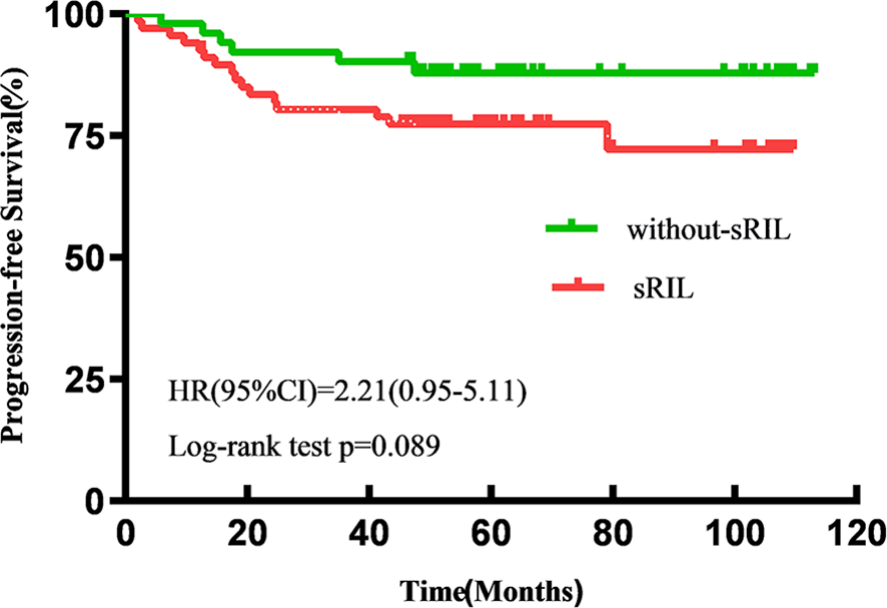
Kaplan-Meier curves of progression-free survival.

With a median follow-up of 57.6 (IQR 47.5-92.8) months, the estimated 5-year OS and PFS for all patients were 87.2% and 82.2%, respectively. Patients with sRIL had significantly decreased OS (79.1% vs 94.1%; HR, 3.81; 95%CI,1.46-9.92; p=0.023, **Figure 1**) and not statistically significant with PFS (76.1% vs 88.2%; HR, 2.21; 95%CI, 0.95-5.11; p=0.089, **Figure 2**) than those without sRIL.

### 3.3 Potential facilitating factors of sRIL

**Figure 3:**
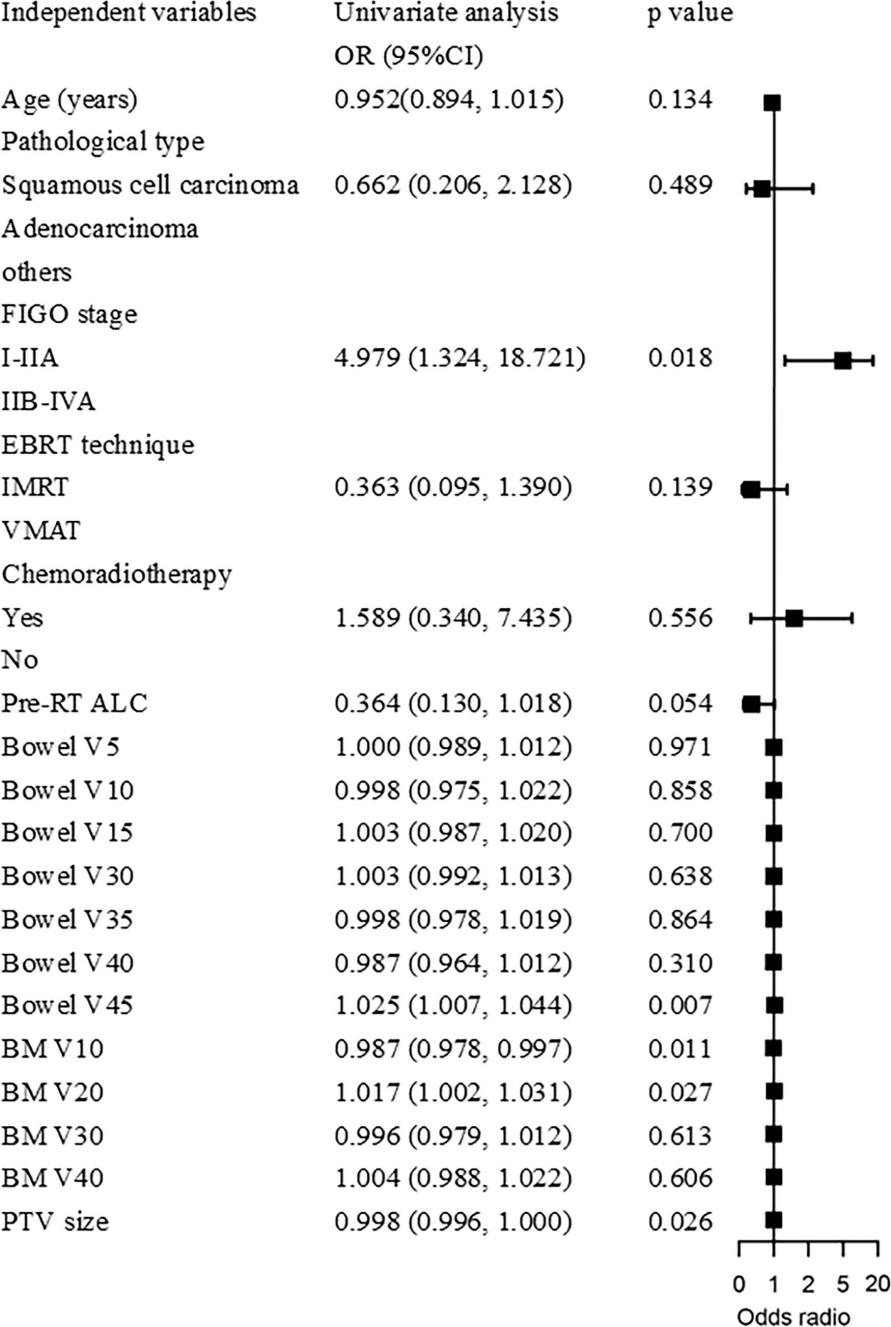
Forest plot of binary logistic regression model for sRIL.

**Figure 4:**
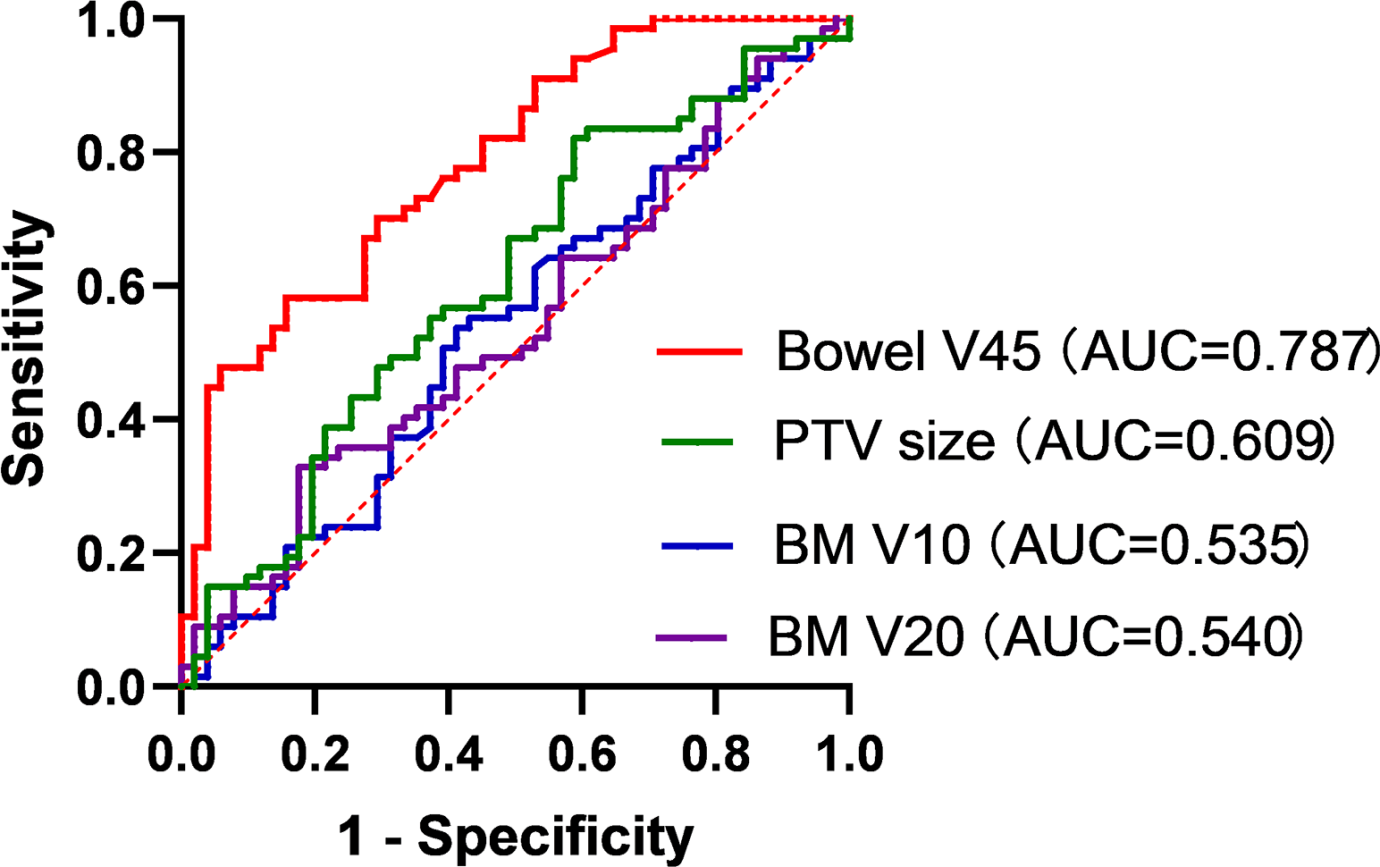
Receiver operating characteristic curve analysis of bowel V45, PTV size, BM V10 and BM V20.

Binary logistic regression analysis revealed that sRIL was significantly correlated with bowel V45 (OR, 1.025; 95%CI, 1.007-1.044; p=0.007), BM V10 (OR, 0.987; 95%CI, 0.978-0.997; p=0.011), and BM V20 (OR, 1.017; 95%CI, 1.002-1.031, p=0.027), and PTV size (OR, 0.998; 95%CI, 0.996-1.000; p=0.026), as shown in **Figure 3**. It was found that bowel V45 (AUC=0.787, p<0.001), rather than PTV size (AUC=0.609, p=0.043), BM V10 (AUC=0.535, p=0.509) or BM V20 (AUC=0.540, p=0.458), was the best indicator for predicting sRIL, as shown in **Figure 4**.

## 4 Discussion

In this study, we found that FIGO stage, concurrent chemotherapy, sRIL after radiotherapy, and pathological type were significant factors affecting OS. Compared to those without sRIL, patients with sRIL showed a significant decrease in OS. Several studies have shown that CC patients with RIL were associated with statistically decreased survival after radiotherapy (22–24). Similar study has found that patients with grade 4 ALC nadir had short OS and PFS (25). This study showed that sRIL did not affect PFS, which could be statistically different if the sample size was enlarged and analyzed again. In addition, immunotherapy, as an emerging therapeutic approach, has the greatest advantage of a long trailing effect after treatment, and therefore has a more significant impact on OS. A study on non-small cell lung cancer showed that sRIL after radiotherapy reduced the survival benefit of CCRT combined with durvalumab (26). Although the precise mechanisms remain unclear, decreased lymphocytes after radiotherapy may contribute to the poor prognosis in CC patients by reducing anti-cancer immunity and increasing the risk of infection (27, 28). Furthermore, the complete adaptive immune system is pivotal for benefiting from immunotherapies (29).

Next, the influences of sRIL were analyzed in the study, and we found that bowel V45, BM V10, BM V20 and PTV size were significant factors. Compared to BM V10, BM V20, PTV size, it was found that bowel V45 was the best indicator for predicting sRIL with ROC curve. This may be the first study to find that bowel dose-volume is a key influencing factor for sRIL. Several studies have found lymphoid organs (nodes, spleen, BM, and thymus in children) may lead to short or continuous lymphopenia (30). In the studies on gastric cancer (31) and pancreatic cancer (32), it was found that the increased radiation dose of the spleen was related to the reduction of lymphocytes after radiotherapy. However, there have been no studies of the relationship between bowel and lymphopenia. As an important lymphoid organ in the body, the bowel plays an integral role in recognizing and responding to foreign pathogens, maintaining mucosal immune homeostasis, and interacting with the host through the microecological environment (33). The gastrointestinal tract contains about 3% of the cells of the immune system and around 5% of the lymphocytes (34). Human gut-associated lymphoid tissues includes both multi-follicular lymphoid tissues, such as Peyer’s patches of the small intestine, and the far more numerous isolated lymphoid follicles (35). The intestinal immune system provides active immunity against invading pathogens, indicating the significant role of lymphocytes within this system (35). Animal studies have found that after FLASH and conventional-dose-rate radiation on the bowel of mice, a significant decrease in the total peripheral blood lymphocyte count can be observed (18). Radiotherapy disrupts the function of the intestinal mucosal immune barrier, which may be the potential mechanism of radiation-induced bowel injury affecting sRIL. And alterations in intestinal immune function are reflected in changes in lymphocyte subtypes in the epithelium and lamina propria, and even further affect peripheral lymphocyte changes (36). This is the first study to demonstrate a correlation between bowel and lymphopenia in CC patients following radiation therapy.

Although, several studies believe that the dose-volume parameters of BM are significantly correlated with RIL (15, 16), there are still conflicting studies on the importance (10, 14). The negative results of the INTERTECC Phase II/III trial may indicate that further exploration is needed regarding the factors that affect sRIL in CC radiotherapy (14). Research has shown that if BMS is too dominant in cervical cancer radiotherapy, BM preservation can cause a significant increase in dose to other critical organs (19, 20). Consequently, the negative result of the INTERTECC Phase II/III trial may be explained as excessive pelvic dose limitation and increased bowel dose exacerbating the decrease in peripheral blood ALC. Reducing BM V10 in CC radiotherapy may help improve RIL, which is agreement with the results of this study (37). During EBRT, both BM and circulating blood cells are exposed to potentially toxic radiation doses, leading to an increased risk of lymphopenia (38). However, there are slight differences in the methods of BM target volume delineation. Most articles define the BM as the entire sacrum, bilateral ilium, ischium, acetabulum, femoral heads up to the ischial tuberosity and vertebral body, and delineate them to ensure reproducibility of clinical procedures (39). Some studies outline the external contours of all bones within PTV, while others use low-density BM gaps or actual proliferating active BM as standards (40). Besides, increased PTV size was correlated with more severe lymphopenia, but was not statistically associated with poor prognosis. Larger PTV increased the radiation dose to the intestine to some extent, further validating our findings.

Radiotherapy technique is crucial for minimizing bowel impact during pelvic radiotherapy. Compared to 3D conventional radiotherapy, IMRT and VMAT can significantly reduce the dose of BM without increasing the dose to bladder, rectum and intestines (41). Although some studies have found that VMAT has advantages in reducing radiotherapy side effects and dose-volume constraints for OAR, more clinical trials are needed to confirm that VMAT is benefit for reducing hematologic toxicity in CC patients (39). Compared to VMAT, proton therapy requires fewer beams to achieve satisfactory dose distribution, high target coverage and sufficient dose-volume constraints for OARs (42). Ongoing advancements in radiotherapy technology and planning may improve patient survival and prognosis. In brief, further clinical trials are essential to understand the mechanism of decreased peripheral blood count and its effect on prognosis.

Due to the varied screening methods of concurrent chemotherapy, there is still considerable controversy over the impact of chemotherapy on RIL. Some studies found that chemotherapy can increase BM toxicity, leading to RIL in CC patients (43). Concurrent chemotherapy and baseline ALC are important clinical factors promoting the lowest point of ALC during radiotherapy (17). On the other hand, the study by Shiraishi Y et al. showed that the more significant decrease of peripheral blood lymphocytes after CCRT rather than during induction chemotherapy (44). Our findings suggest that concurrent chemotherapy is not related to the occurrence of sRIL in CC patients. The lymphopenia after treatment may primarily be caused by ionizing radiation damage to the bowel and BM. In addition, symptomatic supportive treatment during radiotherapy mitigating the effect of chemotherapy and baseline ALC on sRIL. Because the ALCs were collected within one week after radiotherapy rather than at its nadir.

This study has some limitations. First, the retrospective study resulted in inconsistencies blood testing times, complicating the interpretation of the results. Second, the small sample studies may affect the accuracy of predictions. However, we have demonstrated some significant findings, and this study is the first to identify bowel dose-volume as a key influencing factor for sRIL. In clinical practice, implementing stricter constraints to bowel dose-volume during radiotherapy for CC may reduce the incidence of sRIL and further clinical research may be needed to explore the mechanisms involved.

## Data Availability

The raw data supporting the conclusions of this article will be made available by the authors, without undue reservation.
